# Retraction Note: Obesity-induced adipokine imbalance impairs mouse pulmonary vascular endothelial function and primes the lung for injury

**DOI:** 10.1038/s41598-022-06475-2

**Published:** 2022-02-03

**Authors:** Dilip Shah, Freddy Romero, Michelle Duong, Nadan Wang, Bishnuhari Paudyal, Benjamin T. Suratt, Caleb B. Kallen, Jianxin Sun, Ying Zhu, Kenneth Walsh, Ross Summer

**Affiliations:** 1 Center for Translational Medicine and Jane and Leonard Korman Lung Center, Thomas Jefferson University, Philadelphia, PA 19107 USA; 2 Department of Radiology, Thomas Jefferson University, Philadelphia, PA 19107 USA; 3 Department of Medicine, University of Vermont College of Medicine, Burlington, VT 05405 USA; 4 Department of Obstetrics and Gynecology, Thomas Jefferson University, Philadelphia, Pennsylvania 19107 USA; 5 Department of Respiratory Medicine, Changhai Hospital, Second Military Medical University, Shanghai, China; 6 Whitaker Cardiovascular Institute, Boston University School of Medicine, Boston, MA 02118 USA

Retraction of: *Scientific Reports* 10.1038/srep11362, published online 12 June 2015

The Authors have retracted this Article. After publication of this Article, concerns have been raised about irregularities in the western blot data. In particular, the following bands appear to be duplicated:

- Fig. 1e HFD/p-Src lane 1 and 3;
- Fig. 4c NCD/Ve-cadherin lane 1 and 3
- Fig. 5e HFD+ APN/ICAM-1 lane 1 and 2
- Fig. 5f HFD/beta-catenin lane 2 and HFD+ APN/beta-catenin lane 1
- Fig. S1d HFD/beta-catenin all lanes
- Fig. S4c NCD/beta-catenin lane 1 and 3.

Additionally, the beta-catenin subpanel in Fig. 5f was subsequently reused in another study [1] and described as showing GRP87. The Authors were unable to provide the original high resolution scanned images for these blots. Therefore, the validity of the presented results cannot be confirmed.

Dilip Shah, Nadan Wang, Benjamin T. Suratt, Jianxin Sun, Ying Zhu, Kenneth Walsh and Ross Summer agree to this retraction. Freddy Romero, Bishnuhari Paudyal and Caleb B. Kallen have not responded to any correspondence from the editor or publisher about this retraction. The Publisher has not been able to obtain a current email address for Michelle Duong.
